# Pro-mutagenic effects of the gut microbiota in a Lynch syndrome mouse model

**DOI:** 10.1080/19490976.2022.2035660

**Published:** 2022-02-21

**Authors:** Wietske Pieters, Floor Hugenholtz, Kevin Kos, Maxime Cammeraat, Teddy C. Moliej, Daphne Kaldenbach, Sjoerd Klarenbeek, Mark Davids, Lisa Drost, Charlotte de Konink, Elly Delzenne-Goette, Karin E. de Visser, Hein te Riele

**Affiliations:** a Division of Tumor Biology and Immunology, The Netherlands Cancer Institute, Amsterdam, The Netherlands; b Microbiota Center Amsterdam, Amsterdam, The Netherlands; c Oncode Institute, Utrecht, The Netherlands; d Experimental Animal Pathology, The Netherlands Cancer Institute, Amsterdam, The Netherlands

**Keywords:** Lynch syndrome, mismatch repair, microbiota, mutagenesis, colorectal cancer

## Abstract

The gut microbiota strongly impacts the development of sporadic colorectal cancer (CRC), but it is largely unknown how the microbiota affects the pathogenesis of mismatch-repair-deficient CRC in the context of Lynch syndrome. In a mouse model for Lynch syndrome, we found a nearly complete loss of intestinal tumor development when animals were transferred from a conventional “open” animal facility to specific-pathogen-free (SPF) conditions. Using 16S sequencing we detected large changes in microbiota composition between the two facilities. Transcriptomic analyses of tumor-free intestinal tissues showed signs of strong intestinal inflammation in conventional mice. Whole exome sequencing of tumors developing in *Msh2-Lynch* mice revealed a much lower mutational load in the single SPF tumor than in tumors developing in conventional mice, suggesting reduced epithelial proliferation in SPF mice. Fecal microbiota transplantations with conventional feces altered the immune landscape and gut homeostasis, illustrated by increased gut length and elevated epithelial proliferation and migration. This was associated with drastic changes in microbiota composition, in particular increased relative abundances of different mucus-degrading taxa such as *Desulfovibrio* and *Akkermansia*, and increased bacterial-epithelial contact. Strikingly, transplantation of conventional microbiota increased microsatellite instability in untransformed intestinal epithelium of *Msh2-Lynch* mice, indicating that the composition of the microbiota influences the rate of mutagenesis in MSH2-deficient crypts.

## Introduction

Colorectal cancer (CRC) is mostly a sporadic disease, but 5–10% of cases arise as a result of a genetic predisposition of which Lynch syndrome (LS) is the most prevalent.^1^ LS is caused by a heterozygous germline mutation in one of the DNA mismatch repair (MMR) genes, *MSH2, MLH1, MSH6* and *PMS2*.^2^ The MMR pathway corrects base-base mismatches and loops of unpaired nucleotides that arise during DNA replication, thereby strongly repressing spontaneous mutagenesis rates.^3^ Inactivation of the remaining functional allele in somatic cells of mutation carriers disrupts MMR activity, which initiates accumulation of mutations and rapid tumorigenesis. It has been recognized that environmental factors influence CRC development.^4^ In order to effectively design cancer prevention strategies for LS patients, it is essential to dissect the contributions of individual factors to MMR-deficient tumorigenesis.

An important environmental factor is the gut microbiota, the ensemble of bacteria, fungi, archaea, protozoans and viruses that live in close symbiotic relationship with enterocytes, but can also contribute to the development of diseases, including CRC.^5^ Individual microbiota components have been shown to support CRC development and progression via numerous mechanisms.^5^ For instance, it has recently been demonstrated that in a subset of human CRCs, *Pks+ Escherichia coli* derived genotoxins can act on local epithelial cells and deposit a unique mutational signature.^6^ Additionally, indirect mechanisms of microbe-stimulated CRC development have been described, for example, by increased inflammation and proliferation.^5^

While it seems likely that members of the microbiota involved in sporadic CRC development also contribute to the pathogenesis of LS-associated CRC, it is also possible that certain components of the microbiota are specifically hazardous in the context of MMR loss. This is illustrated by experiments in which antibiotic treatment reduced tumor development in *Apc^+/-^;Msh2^−/−^* mice but not in *Apc^+/-^;Msh2^+/-^* mice, suggesting a specific sensitivity of *Msh2*-deficient cells to microbe-mediated oncogenic effects.^7^ In addition, a recent study has prospectively characterized the composition of gut microbiota at taxonomic and functional levels in human LS patients and found increased relative abundance of *Desulfovibrio* to be associated with adenoma development.^8^ However, a cause/consequence relationship could not be established. Therefore, functional studies using *in vitro* and *in vivo* model systems are required to dissect the potential mechanisms of microbial involvement in the pathogenesis of LS-associated CRC.

Here, we studied the role of the microbiota composition on intestinal homeostasis and mutagenesis in our *Lgr5-CreERT2;Msh2^flox/-^ (Msh2-Lynch)* mouse model, in which the tamoxifen-inducible Cre recombinase was expressed by the intestinal-stem-cell-specific *Lgr5* promoter.^9^ LGR5 (Leucine-rich repeat-containing G-protein coupled receptor 5), a receptor for R-spondin and involved in Wnt signaling, is specifically expressed in crypt-base columnar cells.^10^ Hence, in this model, Cre-mediated inactivation of *Msh2* generated a minor number of MSH2-deficient crypts in the intestinal epithelium with increased oncogenic potential.^9^ We previously showed that treatment of *Msh2-Lynch* mice with the methylating drug temozolomide (TMZ) increased the number of MSH2-deficient crypts up to 5-fold, consistent with the fact that MMR deficiency confers resistance to TMZ. Furthermore, TMZ strongly accelerated intestinal tumor development, yielding intestinal tumors in virtually all animals within 4 months.^9^ These observations were made in cohorts of mice that were housed in open cages in a conventional animal facility. Intriguingly, when the mouse line was rederived from the conventional facility into a specific-pathogen-free (SPF) facility with individually ventilated cages, we observed a nearly complete loss of the intestinal tumor phenotype. Transplanting cryo-preserved conventional feces into SPF mice did not restore the tumor phenotype, but did increase epithelial turnover rates and accelerated development of microsatellite instability (MSI). Furthermore, we observed elevated relative abundances of mucus degrading taxa such as *Desulfovibrio* and *Akkermansia*, associated with enhanced epithelial exposure to the microbiota. Our study provides new evidence for a role of the gut microbiota in the development of LS-associated CRC and highlights the importance of using genetic mouse models in different microbial environments in order to elucidate the impact of microbe-stimulated carcinogenesis.

## Results

### Rederivation of *Msh2-Lynch* mice into an SPF facility changes the microbiota composition and strongly suppresses tumor development

**Figure 1.**
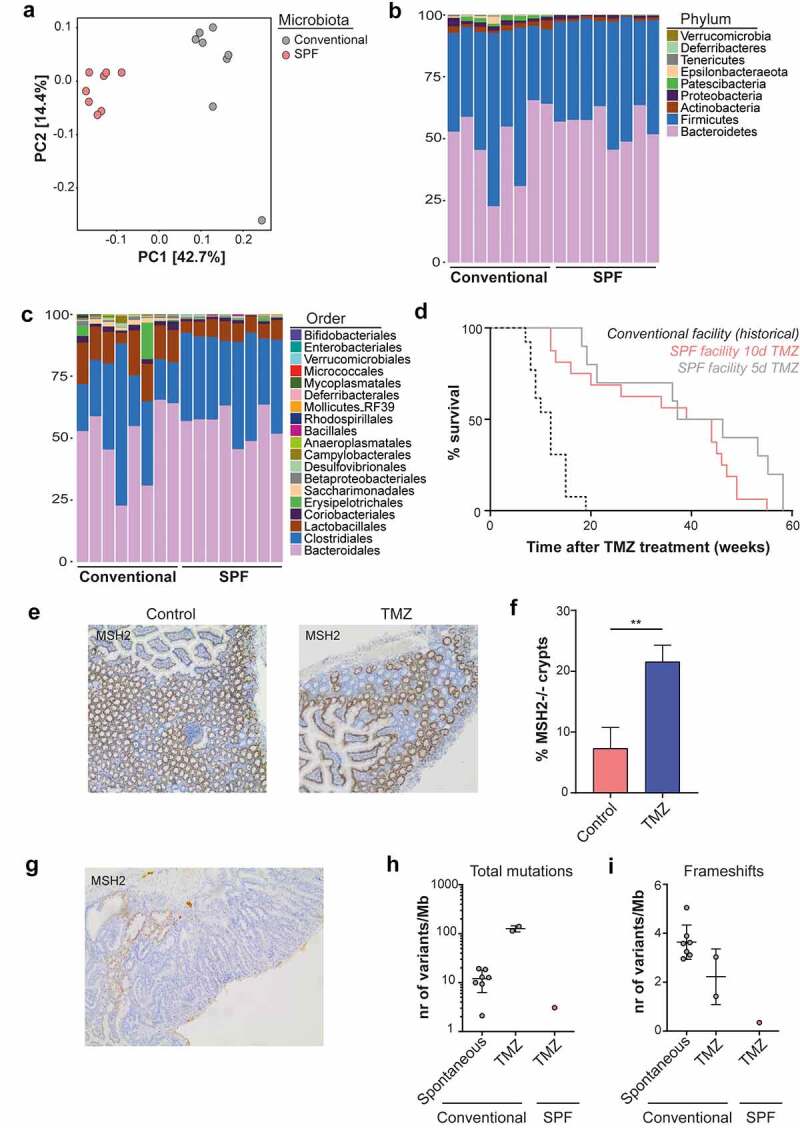
Changes in microbiota composition and tumor phenotypes in *Msh2-Lynch* mice upon rederivation into aSPF facility. The fecal bacterial microbiome was profiled using 16S rRNA gene sequencing (n = 8). A clear distinction between conventional and SPF microbiotas became apparent by: a, Unweighted UniFrac principal component analysis highlighting differences in microbiota composition, b, Relative phylum abundances and c, Relative order abundances. In b and c, each column represents a mouse, 8 from the conventional and 8 from the SPF facility; colors indicate the percentages of different phyla (b) and orders (c) in each animal. d, Tumor-free survival of SPF *Msh2-Lynch* mice after 5d or 10d TMZ treatment (showing no difference: 95% CI of the ratio of median survival = 0.45–2.2, p = .13, log rank test) compared to historical data of conventional *Msh2-Lynch* mice after 10d TMZ treatment^9^ (95% CI of the ratio of median survival of the 10d TMZ-treated cohorts = 1.66–7.19, p < .0001). e, Intestinal MSH2 staining in SPF *Msh2-Lynch* mice without (Control) or after TMZ treatment. f, Percentage of MSH2-deficient crypts in SPF *Msh2-Lynch* mice without (Control, n = 3) or after TMZ treatment (n = 4). Plotted are mean and SD. ** is P < .01 (Student’s t-test). g, MSH2 staining of the single intestinal adenocarcinoma in SPF *Msh2-Lynch* mice. h, Total mutational load and i, frameshift mutations in spontaneously developed (n = 7) or TMZ-induced (n = 2) MMR-deficient intestinal tumors from conventional *Msh2-Lynch* mice (see ref. 9) and the single intestinal tumor in the SPF facility. Mice were treated with TMZ for 5 or 10 d.

Recently, we sanitized and rederived our *Msh2-Lynch* mouse model from a conventional animal facility into a SPF facility. In our conventional facility, mice were housed in open cages, allowing the exchange of commensal and pathogenic microorganisms, while in SPF conditions mice were housed in individually ventilated cages behind a barrier with strict hygiene regimens. Using 16S rRNA gene sequencing, we characterized the fecal microbiota composition in mice from the two facilities. Principal component analysis (PCoA) revealed that microbial profiles of conventional mice clustered separately from SPF mice, indicating mice of the two facilities harbored distinct bacterial communities (Figure 1(a)). Microbiotas from conventional mice showed considerable spread in bacterial composition and bacterial diversity between animals, while SPF microbiotas displayed more uniformity (Figure 1(a-c) and Supplementary Figure S1(a,b)). Assessment of relative taxon abundances demonstrated drastic changes in bacterial microbiota composition between conventional and SPF animals, in which taxa such as *Lactobacillus* and *Epsilonproteobacteria* were strongly reduced or undetected in SPF animals (Figure 1(b,c) and Figure S1(c)). Furthermore, SPF mice were devoid of various viral and parasitic pathogens that were present in conventional animals as detected by commercial pathogen screening (Table S3). Comparison of microbiota compositions of mice housed at different laboratory locations across The Netherlands and from a pet shop revealed that the conventional microbiota was most comparable to microbiota found in the pet shop, which is in line with the open housing environment in the conventional facility (Figure S1(d)).

Our previously published tumor induction experiments in *Msh2-Lynch* mice using the methylating agent TMZ were performed in conventional housing conditions and led to intestinal tumor development in nearly all animals within 4 months.^9^ Strikingly, when we repeated this experiment in SPF conditions, we noticed a nearly complete loss of the intestinal tumor phenotype in SPF conditions, as well as a reduced and delayed incidence of TMZ-induced lymphomas, manifested by a highly significant increase in survival (Figure 1(d) and Table 1). Nonetheless, we could still detect expansion of the MSH2-deficient crypt compartment upon exposure to TMZ in SPF conditions (Figure 1(e,f)) to a similar level as in conventional conditions.^9^ This result underscores that comparison of historical and recent data can be highly misleading, in particular when housing conditions have changed.

**Table 1.** Survival and histopathological analysis of conventional and SPF *Msh2-Lynch* mice after treatment with TMZ

**Housing**	**Case number**	**Survival after TMZ**	**Extraintestinal tumor**	**Intestinal pathology**
Conventional	12HTR244*	7	lymphoid	1,2
Conventional	12HTR249*	8	lymphoid	1
Conventional	13 HTR43*	9		2,3,4
Conventional	12HTR260*	9	lymphoid	2
Conventional	12HTR261*	9	lymphoid	2
Conventional	13 HTR55*	10	lymphoid	2,3
Conventional	13HTR11*	12	lymph/skin	3,4
Conventional	13HTR15*	12	lymphoid	2,3
Conventional	13HTR18*	12	lymphoid	2,3
Conventional	13HTR85*	15	lymph/spleen	2,4
Conventional	13HTR39*	15	lymphoid	
Conventional	13HTR42*	15	spleen	2,4
Conventional	13HTR112*	19		3,4
		**Number of mice: 13**		**Mice with lesions:12**
SPF (5d)	17HTR1	18	lymphoid	
SPF (5d)	17HTR39	19	lymphoid	
SPF (5d)	17HTR56	21	lymphoid	
SPF (5d)	17HTR65	36	lung (adenoma)	4
SPF (5d)	17HTR67	37	lymphoid	
SPF (5d)	17HTR71	46	lung	
SPF (5d)	17HTR128	55	lung	
SPF (5d)	17HTR130	53	lung	
SPF (5d)	17HTR131	58	lung	
SPF (5d)	18HTR7	58	lung	
		**Number of mice: 10**		**Mice with lesions: 1**
SPF (10d)	19HTR87	12	lymphoid	3
SPF (10d)	19HTR88	12	lymphoid	
SPF (10d)	19HTR90	13	lymphoid	
SPF (10d)	19HTR104	16	lymphoid	
SPF (10d)	19HTR114	20	lymphoid	
SPF (10d)	19HTR128	26	lung	
SPF (10d)	19HTR163	34	genital tract	2
SPF (10d)	19HTR190	39	lung	
SPF (10d)	19HTR208	44	lymphoid	
SPF (10d)	19HTR215	44	lung	
SPF (10d)	19HTR217	45	lung	
SPF (10d)	19HTR225	46	lung	
SPF (10d)	19HTR227	47	lung	
SPF (10d)	19HTR228	49	lung	
SPF (10d)	19HTR229	49	lung	
SPF (10d)	20HTR34	55	lung	
		**Number of mice: 16**		**Mice with lesions: 2**

Survival after TMZ is in weeks; Intestinal pathology: 1, focal hyperplasia; 2, gastrointestinal neoplasia (GIN); 3, adenoma; 4, adenocarcinoma; *: cohort of published study.^9^

Conventional 10d TMZ *versus* SPF 5d TMZ: p = 0.000345.

Conventional 10d TMZ *versus* SPF 10d TMZ: p = 0.000066.

(Fisher’s exact test with Bonferroni correction).

In order to gain insights in potential mechanisms that govern the reduction in intestinal tumorigenesis in SPF *Msh2-Lynch* mice, we performed whole exome sequencing on the single intestinal tumor that we obtained in the SPF facility and on tumors that arose spontaneously or in response to TMZ in conventional *Msh2-Lynch* mice as described in Wojciechowicz *et al*. (2014).^9^ Mutational analysis of the tumors that developed in TMZ-treated, conventionally housed *Msh2-Lynch* mice revealed a high total mutational burden, as would be expected (Figure 1(h)). In contrast, the mutational load in the sole intestinal tumor that arose in our TMZ-treated SPF cohort was dramatically lower (Figure 1(g,h)). Furthermore, the number of frameshift mutations was also strongly reduced in the SPF tumor (Figure 1(i)). We realize that this observation in a single tumor bears no statistical significance. Nonetheless, as frameshift mutations accumulate independently of TMZ treatment but only as a result of MMR deficiency, this could be reflective of reduced epithelial turnover rates. Alternatively, reduced mutational load in this single tumor and total absence of tumor development in the rest of the cohort may be caused by the active elimination of neoantigen-expressing cells by the adaptive immune system in SPF mice. To test the latter hypothesis, we crossed our *Msh2-Lynch* mouse model into a *Tap1^−/−^* background, which is deficient for the TAP1 transporter required for antigen loading into MHC-I, and consequently lacks CD8 + T cells.^11^ *Tap1^−/−^Msh2-Lynch* mice in SPF conditions did not show intestinal tumor development nor differences in survival or in the percentage of MSH2-deficient crypts after TMZ treatment as compared to TAP1-proficient animals, arguing against immunosurveillance in SPF mice (Figure S1(e,f)).

Together, our data suggest that the intestinal microbiota composition may contribute to MMR-deficient tumorigenesis by influencing the rate of intestinal cell turnover.

### Intestines of conventional and SPF mice show different transcriptional profiles

**Figure 2.**
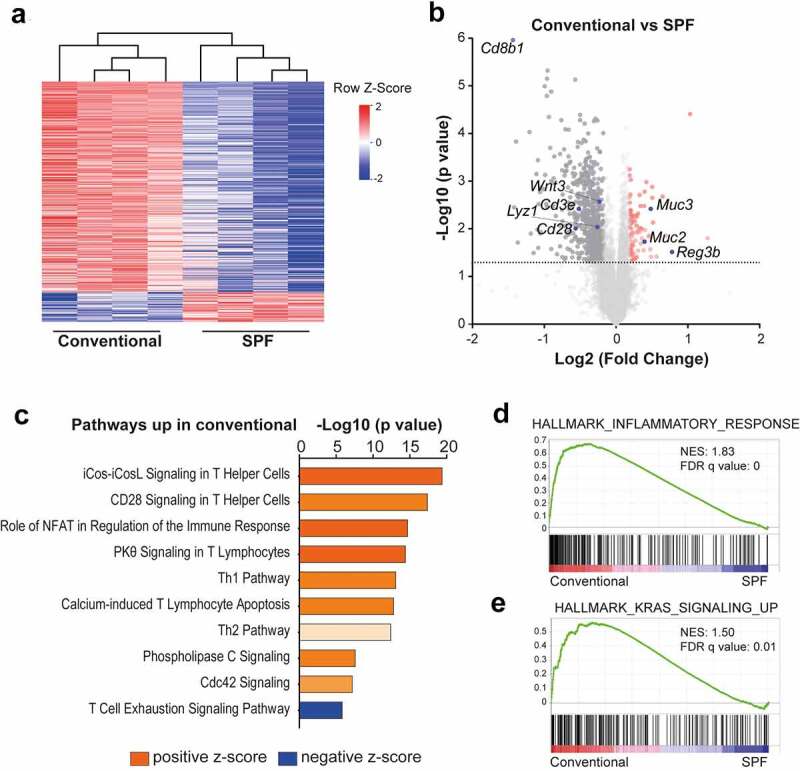
Intestines of conventional and SPF mice show strongly divergent transcriptional profiles. RNA sequencing was performed on −80°C preserved tumor-free small intestines from tamoxifen-treated conventional or SPF *Msh2-Lynch* mice (n = 4). a, Heatmap depicting row z-scores of and b, Volcano plot, showing differentially expressed genes (P < .05, FC>1.5). c, IPA on differentially expressed genes between SPF and conventional mice. The top 10 significant pathways with positive or negative z-score are shown. d, GSEA analyses comparing transcriptional profiles of conventional and SPF mice to MSigDB Hallmark gene sets. Normalized enrichment score (NES) and false discovery rate (FDR) q-value are indicated.

To evaluate whether and how an altered microbial environment affects intestinal tissue, we extracted RNA from −80°C preserved crude small intestines derived from tumor-free conventional and SPF *Msh2-Lynch* mice and performed RNA sequencing. Unsupervised hierarchical clustering of differentially expressed genes revealed numerous changes in gene expression patterns (Figure 2(a)). Intestines of conventional mice mainly showed enhanced expression of immune related genes but remarkably also increased abundance of *Lyz1* and *Wnt3* transcripts, indicative of elevated Wnt production by Paneth cells that stimulates intestinal stem cell proliferation (Figure 2(b)).^12^ Conversely, SPF mice showed increased expression of mucins *Muc2* and *Muc3* and the antimicrobial peptide *Reg3b*, which function to protect the intestinal epithelium from direct contact with the intestinal microbiota (Figure 2(b)).^13,14^

Ingenuity pathway analysis (IPA) revealed upregulation of predominantly T-cell-mediated inflammatory pathways in conventional animals (Figure 2(c) and Figure S2(a)). In line with this, gene set enrichment analysis (GSEA) using the 50 MSigDB Hallmark Gene sets showed a strong enrichment of transcriptional signatures related to inflammatory responses in intestines of conventional mice (Figure 2(d) and Figure S2(b)). Of note, two of the other significantly enriched gene sets in conventional animals were K-Ras signaling and epithelial to mesenchymal transition, which have been associated with cancer development and progression (Figure 2(e) and Figure S2(b)).

The cumulative results of our transcriptional analyses demonstrate that intestines of conventional mice were in an inflamed state and showed upregulation of cancer promoting signaling pathways.

### Transplantation of conventional microbiota changes intestinal homeostasis

**Figure 3.**
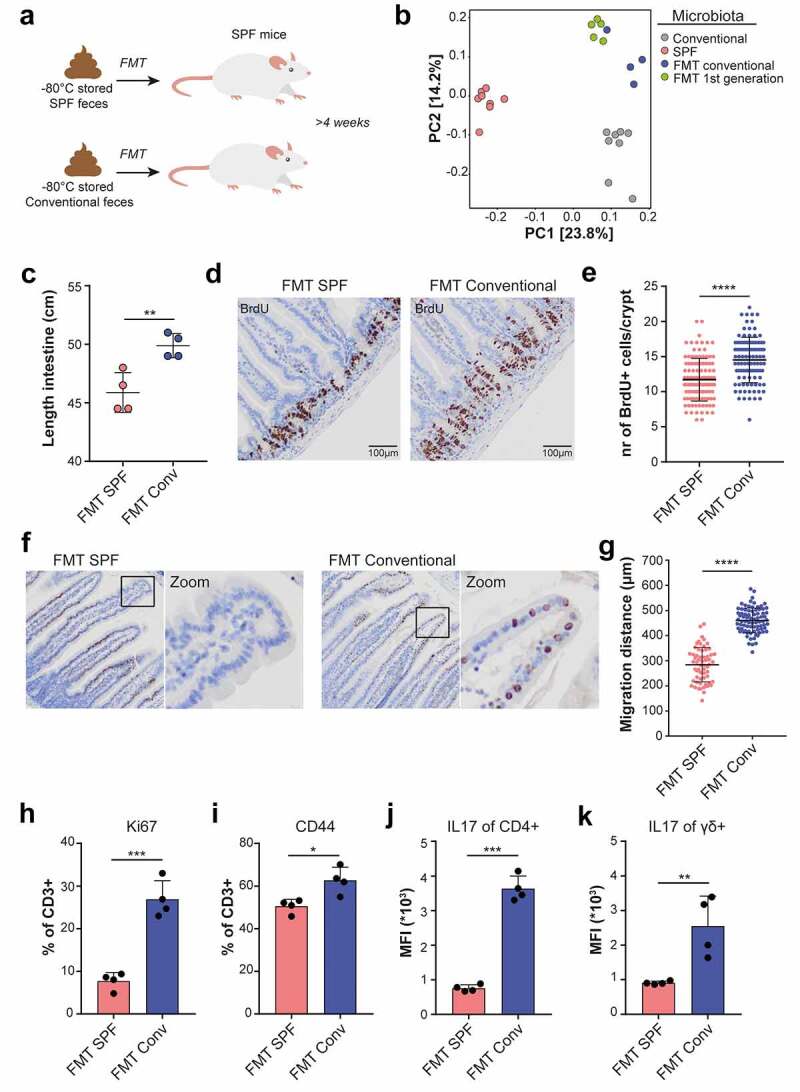
Transplantation of conventional microbiota alters intestinal homeostasis. a, Experimental setup. FMT experiments were performed in WT mice. b, Unweighted UniFrac PCoA displaying alterations in bacterial microbiota compositions after conventional FMT or in the first generation offspring, based on 16S rRNA gene sequencing. c, Quantification of small intestinal length after FMT (n = 4). d, Jejunal BrdU staining 2 hours after BrdU injection at 8 weeks after FMT, isolated. e, Quantification of d. (n = 3) f, Jejunal BrdU staining 2 d after BrdU injection at 4 weeks after FMT. g, Quantification of the distance from the start of the villus to the foremost BrdU positive in 20 well-oriented villi per mouse (SPF n = 3, conventional n = 4). h-k, T cell composition in LPL after FMT (n = 4). *(h)* Ki67+ and *(i)* CD44+ gated on live, CD45+, CD3 + T cells. j,k, Median fluorescence intensity (MFI) for IL17 in LPL T cells after FMT, gated on j, live CD45+, CD3+, γδ-, CD4+ and K, CD45+, CD3+, γδ+ cells. Plotted are mean and SD. Asterisks indicate a significance level of P < .05, P < .01, and P < .001, respectively (Student’s t-test).

We next assessed whether the microbiota and phenotypic differences between conventional and SPF conditions are causally related. Unfortunately, the dismantling of the conventional facility prohibited us from using fresh fecal material. However, we had access to −80°C preserved conventional feces that we used in fecal microbiota transplantations (FMT) into SPF mice, realizing that not all components may have survived the freezing procedure (Figure 3(a)). 16S rRNA gene sequencing of fecal pellets derived from mice that received FMT, or their offspring (FMT 1st generation), revealed that conventional FMT stably altered the composition of the bacterial gut microbiota. However, the microbiota composition remained distinct from that of the original conventional mice, which is likely caused by loss of certain bacterial taxa due to suboptimal storage conditions of the fecal pellets (Figure 3(b)). Next to changes in bacterial microbiota composition, mice that underwent conventional FMT tested positive for a selection of pathogens that were originally present in mice of the conventional facility (Table S3).

Although we failed to fully reconstitute the original microbiota, we made some remarkable observations. First, mice that received conventional FMT showed a significant lengthening of the small intestine as compared to SPF transplanted controls (Figure 3(c)). This effect was accompanied by elevated epithelial proliferation as measured by an increase in the number of 5-bromo-2’-deoxyuridine (BrdU) incorporating cells per intestinal crypt (Figure 3(d,e)). Similar observations were made in the offspring of mice that received conventional FMT, indicating that these intestinal responses were evoked by viable and transmissible microbiota components and not by passive metabolites that were present in conventional feces (Figure S3(a,b)). Second, the migration distance of BrdU+ cells along the villus axis, measured 2 d after BrdU injection, was significantly elevated in animals that had received conventional FMT, indicative of a shorter epithelial transit time (Figure 3(f,g)). Third, using flow cytometry, we profiled intestinal T cell responses upon FMT in intraepithelial lymphocytes (IEL) and lamina propria lymphocytes (LPL). Intestinal IEL- and LPL-derived T cells from animals that received conventional FMT showed increased percentages of Ki67+ and CD44+ cells as compared to SPF transplanted animals, indicative of enhanced T cell proliferation and activation (Figure 3(h,i) and Figure S3(c,d)). In the LPL compartment, both CD4+ and γδ+ T cells showed increased levels of IL17 and a trend was observed toward increased percentages of IFN-γ+ CD8 + T cells, all indicative of T-cell mediated inflammatory responses (Figure 3(j,k) and Figure S3(e)).

In summary, these results demonstrate that transplantation of −80°C preserved conventional feces restored viable components of the microbiota, accelerated epithelial proliferation and migration, and evoked inflammatory immune responses.

### Conventionalized mice show increased abundance of mucus associated taxa

We sought to identify microbial factors in the conventional microbiota that promote intestinal proliferation rates. Since mice that received conventional FMT harbored viral pathogens including mouse parvovirus (MPV) that has been shown to infect enterocytes, we first explored the potential role of viral factors on epithelial proliferation (Table S3).^15,16^ For that matter, we performed FMT using a conventional fecal suspension that was passed through a 0.22 µm filter to selectively obtain the viral fraction of the microbiota (Figure S4(a)). Similar to conventional FMT, treatment of mice with the filtered fecal suspension effectively established MPV infection (Figure S4(b)). However, administration of filtered conventional feces failed to induce epithelial proliferation or gut lengthening, indicating that epithelial responses evoked by the conventional microbiota were not solely induced by a virus (Figure S4(c,d)).

**Figure 4.**
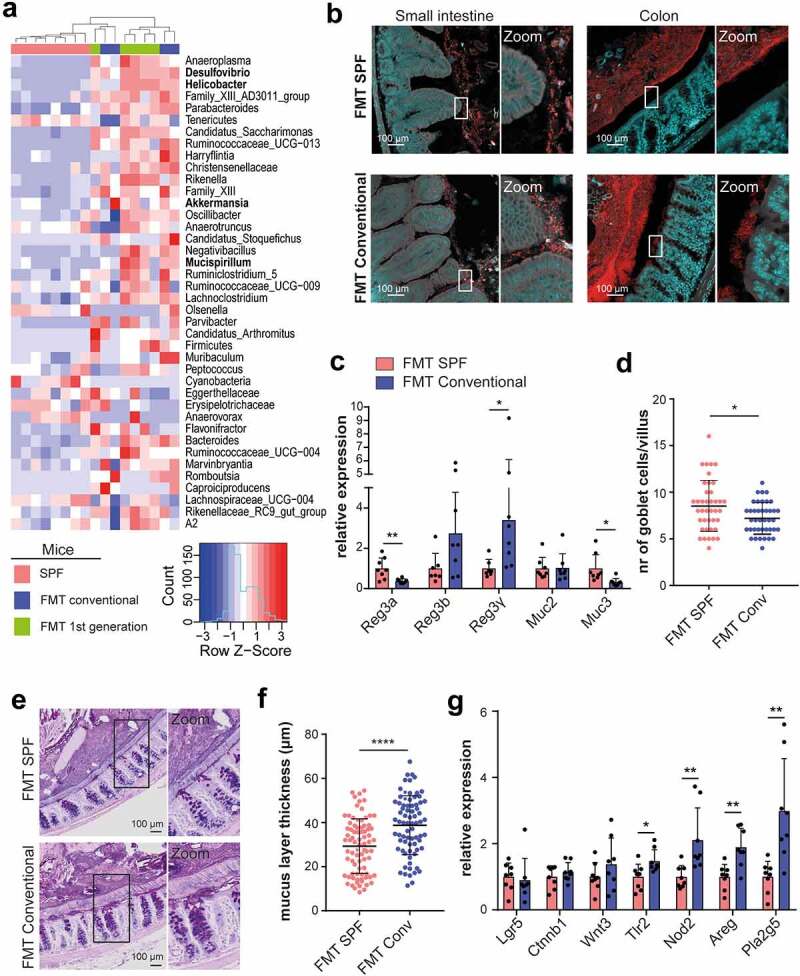
The conventional microbiota invade the intestinal mucus layer and evoke proliferative epithelial responses. a, Heatmap depicting row z-scores of bacterial genera that showed significant changes (P < .05) in relative abundance between SPF mice that underwent conventional FMT and the first generation offspring. Genera known to reside within or in close proximity to the intestinal mucus layer and epithelial border are indicated in bold. b, Images of small intestine and colon derived from WT mice at 4 weeks after SPF or conventional FMT stained with Cy-3 labeled pan-bacterial rRNA FISH probe mix (EUB338-I,II and III) and DAPI c, Relative intestinal expression of antimicrobial and mucin genes after SPF or conventional FMT (n = 8). d, Number of goblet cells per villus as quantified from Alcian Blue-Periodic Acid Schiff (AB-PAS) stained jejunal tissue at 4 weeks after SPF or conventional FMT (n = 4). 10 villi per mouse were analyzed. e, Colonic tissues derived from WT mice at 4 weeks after SPF or conventional FMT stained with AB-PAS at pH 1.5 to visualize the mucus layer. f, Quantification of mucus layer thickness from d. The thickness of the colonic mucus layer was measured in 20 areas across entire transversal colonic sections (n = 4). g, Relative gene expression levels in small intestinal crypts after SPF or conventional FMT (n = 8). Plotted are mean and SD. Asterisks indicate a significance level of P < .05 and P < .01, respectively (Student’s t-test) .

We therefore further explored the alterations in bacterial microbiota composition upon conventional FMT. 16S rRNA gene sequencing revealed that among the genera that were significantly enriched in mice that received conventional FMT and their offspring were border dwelling taxa that have been found to reside in close proximity to the intestinal mucus layer, including *Desulfovibrio, Helicobacter, Akkermansia* and *Mucispirillum* (Figure 4(a)).^17–20^ *Desulfovibrio* and *Akkermansia* are active mucin degraders, which could increase mucus layer permeability and promote direct interaction of border dwelling taxa with the intestinal epithelium.^17,18^ Using panbacterial 16S rRNA fluorescence in situ hybridization (FISH) probes, we assessed bacterial localization relative to the epithelium in both small and large intestinal tissues of animals that received SPF or conventional FMT. Interestingly, in mice that received conventional FMT, the interaction between bacteria and the villus tips, as well as the presence of bacteria between the villi were significantly increased as compared to mice that received SPF FMT (Figure 4(b) and Figure S5(a,b)). Correspondingly, intestines of conventional FMT mice showed altered expression levels of antimicrobial peptides *Reg3a,b* and *γ* and *Muc3*, indicative of epithelial responses to the microbiota (Figure 4(c)). In line with this, the number of mucus-producing goblet cells in the small intestine was reduced in conventional FMT mice (Figure 4(d)). A similar, though non-significant trend was seen in the colon: conventional FMT mice demonstrated increased bacterial invasion into the mucus layer and bacterial adhesion to epithelial cells, despite the presence of a thicker colonic mucus layer (Figure 4(b,e,f) and Figure S5(c,d)).

To assess whether the increased epithelial turnover levels in conventional FMT mice were mediated by adaptive immune responses against invading members of conventional microbiota, we repeated the FMT protocol in *Rag1^−/−^* mice, which are devoid of mature T and B cells.^21^ Interestingly, the proliferative response of the epithelium upon conventional FMT was aggravated in *Rag1^−/−^* mice (Figure S6(a)). Furthermore, compared to WT mice, *Rag1^−/−^* mice receiving SPF FMT showed enhanced bacterial invasion (compare Figures S6(b) and S5(a); left panels, p = .0374, Chi-square test), as well as epithelial adhesion (compare Figures S6(c) and S5(b); left panels, p = .0254, Chi-square test) and for bacterial invasion, this was exacerbated upon conventional FMT (compare Figures S6(b) and S5(a); right panels). In the colon, no statistically significant differences (Fisher’s exact test) between transplanted *Rag1^−/−^* and WT mice were seen, but also in *Rag1^−/−^* mice a trend was seen for a slightly more potent effect of conventional FMT (compare Figure S6(d,e) and S5(c,d)). Thus, it seems that adaptive immune responses do not mediate, but rather counteract microbiota-induced epithelial proliferation, as well as microbial invasion into the small intestinal epithelium, suggesting that these two processes are related.

Intestinal epithelial cells have been shown to express several types of innate pattern recognition receptors that, upon binding with their respective microbe-associated molecular pattern (MAMP) such as flagellin or lipopolysaccharide (LPS), trigger a range of cellular responses, including proliferation.^22,23^ We therefore evaluated the expression levels of pattern recognition receptors and downstream targets involved in epithelial maintenance and repair in small intestinal crypts derived from mice that received SPF or conventional FMT. Conventional FMT did not alter the expression of Wnt-related genes (Figure 4(g)). However, intestinal crypts derived from conventional FMT mice showed elevated expression of *Tlr2* and *Nod2*, as well as pro-proliferative transcripts *Pla2g5* and *Areg*, the latter encoding for the epithelial growth factor receptor (EGFR) ligand Amphiregulin (Figure 4(g)).^24^

Combined, our data indicate that mucus-degrading members of the conventional microbiota alter the permeability of the intestinal mucus layer, which may facilitate epithelial exposure to other luminal microbiota components as well and trigger the activation of innate pro-proliferative pathways in epithelial cells.

### Impact of the conventional microbiota on intestinal tumorigenesis and mutagenesis

**Figure 5.**
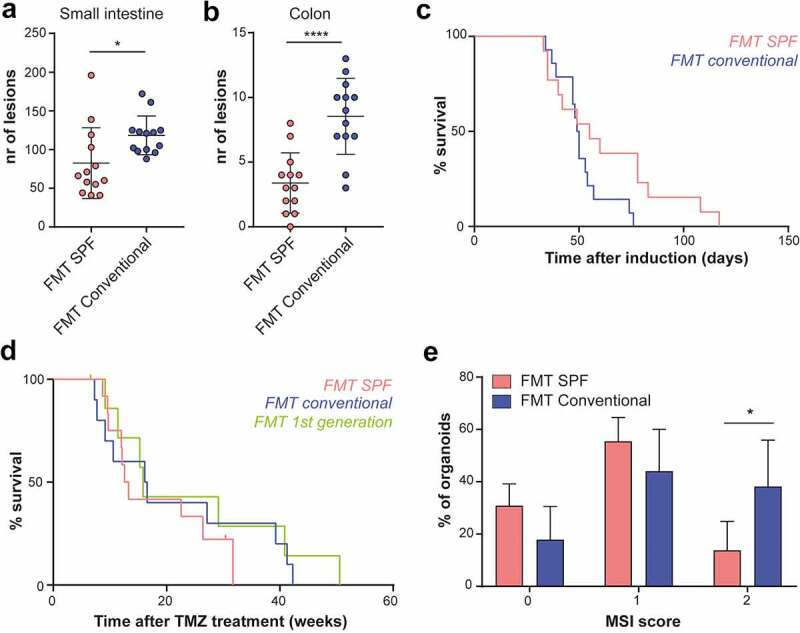
The conventional microbiota differentially impact tumorigenesis and mutagenesis in distinct CRC models. a, Number of lesions scored from HE stained small intestinal tissue sections of *Lgr5-Cre;Apc^flox/flox^* mice after SPF or conventional FMT (n = 13). Neoplastic lesions up to a size of 100 µm were included in the analysis b, Number of colonic lesions. Plotted are mean and SD. Asterisks indicate a significance level of P < .05, P < .01, P < .001 and P < .0001, respectively (Student’s t-test). c, Survival after induction of *Apc* loss in SPF- or conventional-FMT-treated *Lgr5-Cre;Apc^flox/flox^* mice (n = 13). d, Survival after 10d TMZ treatment of *Msh2-Lynch* mice that received SPF or conventional FMT or born as the first generation after conventional FMT. e, Distribution of the MSI score in small intestinal MSH2-deficient organoids derived from *Msh2-Lynch* mice after SPF (n = 4) or conventional (n = 5) FMT. Plotted are mean and SD. Asterisk indicates a significance level of P < .05 (Two-way ANOVA, with Sidak’s test for multiple comparisons).

In order to investigate whether conventional FMT could impact intestinal tumorigenesis, we first used a classical CRC model, *Lgr5-CreERT2;Apc^flox/flox^*, in which tumorigenesis can be induced by homozygous deletion of *Apc* in intestinal stem cells, leading to numerous lesions across the intestinal tract. FMT with conventional microbiota strongly increased the number of lesions, both in the small intestine (Figure 5(a)) and in the colon (Figure 5(b)), without significantly impacting survival (p = .08) (Figure 5(c)).

We next questioned whether the conventional FMT restores intestinal tumorigenesis in *Msh2-Lynch* mice. Cohorts of *Msh2-Lynch* mice that had received SPF or conventional FMT or were born from conventional FMT mice were monitored for tumor incidence after treatment with TMZ. In this model, conventional FMT did not alter survival or intestinal tumor incidence (Figure 5(d) and Table S4).

Nonetheless, the enhanced epithelial turnover imposed by the microbiota conceivably accelerates the accumulation of mutations in MSH2-deficient crypts. As a readout for spontaneous mutagenesis, we quantified MSI levels in MSH2-deficient stem cells exposed to different microbial conditions for defined time-periods. To obtain sufficient cell numbers for MSI analysis, we isolated intestinal crypts from FMT-treated *Msh2-Lynch* mice and cultured them in the presence of the methylating agent 6-thioguanine (6-TG) to selectively obtain MSH2-deficient organoids. These were individually analyzed for the presence of MSI by PCR. Strikingly, conventional FMT significantly elevated MSI levels in MSH2-deficient organoids, as compared to FMT with SPF feces, indicating that the rate of mutagenesis in MSH2-deficient intestinal crypts is influenced by the composition of the microbiota (Figure 5(e)).

Together, our results indicate that conventional microbiota components promote intestinal tumor development due to *Apc* loss. In *Msh2-Lynch* mice, enhanced epithelial turnover rates did not restore intestinal tumorigenesis but increased the rate of spontaneous mutagenesis in MSH2-deficient crypts, indicating that the microbiota contributes to a key step in cellular transformation of MMR-deficient cells.

## Discussion

Insights into the role of external factors in the pathogenesis of CRC are crucial for the development of cancer prevention strategies, which particularly benefit genetically predisposed individuals such as LS patients. There is an emerging interest in the role of the microbiota in CRC development as the microbiota composition can be manipulated relatively easily by factors such as diet and pre-, pro- or antibiotic treatments. Although several mechanisms have been proposed to explain the impact of the gut microbiota on intestinal tumor development, it remains largely unknown whether and how the gut microbiota specifically affects MMR-deficient tumorigenesis. Using our *Msh2-Lynch* mouse model, we studied the role of the gut microbiota on intestinal homeostasis and tumor development. We observed that the rederivation of our *Msh2-Lynch* mouse line from a conventional facility into an SPF facility was accompanied with strong changes in microbiota composition and a nearly complete loss of the intestinal tumor phenotype. The outcomes of our initial analyses on both healthy intestinal tissue and tumor samples from conventional and SPF *Msh2-Lynch* mice led us to hypothesize that SPF mice display reduced intestinal epithelial turnover, which would diminish the accumulation of mutations in MMR-deficient crypts. We found that partial restoration of the conventional microbiota composition augments intestinal cell turnover as evidenced by enhanced proliferation and migration and thereby the rate by which mutations accumulate in MSH2-deficient crypts.

Since the discovery of bacteria residing within or in close proximity to tumors, several individual species have been identified to impact CRC development, such as *Pks+ E. coli*, or *Fusobacterium nucleatum*.^6,25,26^ Interestingly though, a prospective study in LS patients found that adenoma development was not associated with these well-studied oncomicrobes but was rather associated with microbiota components that broadly influence gut microbial ecology.^8^ Our results support a scenario in which global changes in epithelial homeostasis, evoked by shifts in the gut microbiota composition, contribute to the accumulation of mutations in MSH2-deficient crypts. Others have shown that the bacterial metabolite butyrate can induce hyper-proliferation specifically in MSH2-deficient colonic crypts by enhancing Wnt/β-catenin signaling.^7^ As in our study, increased epithelial proliferation was detected throughout the small intestine and also in WT animals, we consider it unlikely that these effects are evoked by the same mechanism.^7^ Nonetheless, future studies are needed to determine whether bacterial metabolites directly impact intestinal cell turnover and specifically enhance mutagenesis in MSH2-deficient crypts.

The results of our study indicate that bacterial invasion of the mucus layer and interaction with the epithelium may contribute to early steps of MMR-deficient cellular transformation by elevating epithelial proliferation rates. Bacterial invasion into the intestinal mucus layer is a commonly observed phenomenon in patients suffering from inflammatory bowel disease (IBD), but also occurs in other pathological conditions that involve bacterial dysbiosis.^27,28^ The presence of bacteria in tumor crypts has been observed in *Villin-Cre;Msh2^lox/lox^;IL10^−/−^* mice, but it remained unclear whether this was a cause or a consequence of tumor development.^29^ We found that the increased bacterial presence in the mucus layer and enhanced proliferation rates are associated with increased relative abundances of mucus colonizing taxa, such as *Akkermansia, Mucispirillum* and *Desulfovibrio*, as has also been observed by others.^30^ Notably, increased abundance of *Desulfovibrio* has been associated with adenoma development in LS patients.^8^ We do not know whether bacterial mucus invasion is confined to mucus-degrading bacteria only or whether dilation of the mucus layer (perhaps leading to the observed thickening of the mucus layer) allows access to other bacteria as well. E.g., next to potential tissue damaging effects by the reduction of sulfide, *Desulfovibrio* may enhance the permeability of the mucus layer, and thereby indirectly promote epithelial proliferation due to increased epithelial exposure to the luminal microbiota.^30,31^

An association between gut microbiota, inflammation and tumorigenesis in the context of *Apc* loss has been reported previously, and our observation that the conventional microbiota enhances tumorigenesis in *Lgr5-Cre;Apc^flox/flox^* mice therefore adds to mounting evidence.^32^ We show that conventional FMT strongly induces IL17 production in lamina propria T cells (Figure 3(h,i)) in WT mice, and this cytokine has been shown to promote tumorigenesis throughout the intestinal tract, which matches our observations that conventional FMT enhances both small intestinal and colonic tumorigenesis in *Lgr5-Cre;Apc^flox/flox^* mice. Furthermore, we observed that FMT with conventional feces leads to increased relative abundances of *Helicobacter* and *Akkermansia*, and the combined presence of these taxa has been linked to increased tumorigenesis in *Apc* mutant mice.^20^

While exposure to the conventional microbiota increased tumor incidence in *Lgr5-Cre;Apc^flox/flox^* mice, it did not restore the intestinal tumor phenotype in *Msh2-Lynch* mice. This may be explained by the fact that we were able to only partially recover components of the conventional microbiota. Using 16S rRNA gene sequencing and commercial pathogen screening on FMT mice, we show that the microbiota composition became more comparable to that of the conventional facility, but did not return to its original state (Figure 3(b) and Table S3). It may be that certain components of the microbiota that are lacking after conventional FMT play a crucial role in tumor development in conventional *Msh2-Lynch* mice. For example, conventional mice showed infection with various nematode species, and epithelial hyper-proliferation is part of the nematode expulsion cycle.^33^ Despite our observations that conventional FMT did increase epithelial turnover, it could be that turnover rates were still drastically lower than in mice housed in the original conventional facility. This is also in line with the observation that intestines of these conventional mice displayed increased expression of Wnt3, which promotes stem cell proliferation, survival and tumor development, while Wnt3 expression was not elevated upon conventional FMT (Figures 2(b) and 4(f)).^34^ To obtain a broader reconstitution of the intestinal phenotypes observed in conventional mice, it would be highly relevant to perform FMT experiments with microbiota derived from pet shop mice, as the microbiota composition of these mice is most comparable to that of conventional mice (Figure S1(d)). However, institutional regulations have thus far prohibited us from performing these experiments. Finally, we cannot fully exclude that genetic alterations arose during the rederivation process of our *Msh2-Lynch* mouse line that may have contributed to the loss of intestinal tumor development in the SPF facility, although we found no evidence for this by whole exome sequencing.

In conclusion, we demonstrated here that the microbiota strongly impacts the inflammatory state of the intestine, mucus layer homeostasis, the rate of epithelial turnover and mutagenesis in MSH2-deficient crypts. These observations corroborate the worry that the strict sanitary standards that are adopted by many animal facilities may mask interesting and relevant phenotypes in studies aimed at modeling and understanding human disease. Given the general nature of our observations, we anticipate that these effects also occur in humans, although the exact causative agents may differ. Identification of these factors may eventually allow the development of CRC prevention strategies that target the microbiota.

## Materials and methods

### Mice and treatments

Mice were housed at a relative humidity of 55% in individually ventilated cages (Innovive®). FMT experiments were performed in isolators. Mice were fed Transbreed (E) PL MIN pellet nutrition (Special Diet Services) and water (Aquavive®) *ad libitum. Lgr5-CreERT2;Msh2^flox/-^* (*Msh2-Lynch), Tap1^−/−^;Msh2-Lynch,Rag1^−/−^*, and *WT* mice were on the FVB background. *Lgr5-Cre;Apc^flox/flox^* were on the C57/BL6 background. Animals were genotyped using allele-specific primers. Induction of the *Msh2-Lynch* mouse model by tamoxifen and subsequent TMZ treatments were conducted as described.^9^ *Lgr5-Cre;Apc^flox/flox^* mice received intraperitoneal (IP) injection with tamoxifen (120 mg/kg). Unless stated otherwise, mouse lines were maintained in SPF conditions as listed in Supplementary Table S1. Pathogen screening was done by IDEXX. Short-term experiments were performed using female mice. In tumor cohorts and MSI experiments, both sexes were used. Mice were sacrificed using CO_2_ or by cervical dislocation. All experiments were performed in accordance with Dutch and European guidelines and were approved by the local Animal Ethical Committee at The Netherlands Cancer Institute and the National Commission for Animal Experimentation (Centrale Commissie Dierproeven) of The Netherlands.

### 16S rRNA gene sequencing

Similar to conventional mice, fecal pellets from SPF mice, FMT animals, mice housed at VU, Radboud, Erasmus, Groningen and Utrecht University or the pet shop (Parva, Leiden, The Netherlands) were stored at −80°C until further processing. Bead-beating of the pellets was performed as described, supplemented with Stool transport and recovery (S.T.A.R.) buffer (Roche).^35^ DNA was extracted using the Maxwell RSC Blood DNA Kit (Promega). The V3-V4 region of the 16S rRNA gene was amplified, with barcoded 341 forward and 805 reverse primers was purified using AMPure XP beads (Beckman Coulter) and loaded on the MiSeq with the MiSeq V3 – 600 cycle kit (Illumina).^36^ The forward and reverse reads were length trimmed at 240 and 210, respectively, inferred and merged with ASVs using DADA2 V.1.5.2. Assignment of taxonomy was done using the DADA2 implementation of the RDP classifier and SILVA 16S reference database.^37,38^ Further statistical tests were performed using the phyloseq and microbiome package in R.

### Fecal microbiota transplantations

Fecal pellets (-80°C stored) were suspended in PBS and filtered using a 100 µm strainer. Mice received 300 µl of the fecal suspension by oral gavage. *Msh2-Lynch* and *Lgr5-Cre;Apc^flox/flox^* mice, animals were pre-treated with antibiotics ampicillin (1 mg/ml), streptomycin (5 mg/ml), and colistin (1 mg/ml) in the drinking water for 3 consecutive days before 10 daily FMTs. When we noticed that 3 daily FMTs, without antibiotic pre-treatment sufficed to induce the described phenotypes, this regimen was implemented in all other experiments. Phenotypes were examined at least 4 weeks after FMT. For mouse MPV detection, fecal DNA was extracted using DNeasy PowerSoil Kit (Qiagen), and PCR was performed using MPV specific primers (Supplementary Table S2).

### Immunohistochemistry and Histopathologic analysis

Intestines were fixed in 4% formaldehyde, processed into Swiss rolls, and embedded in paraffin.^39^ Sections were stained with H&E or stained for MSH2.^9^ Mice were IP injected with 1 mg of BrdU in PBS and intestines were collected 2 h or 2 d later. Sections were stained with anti-BrdU antibody (Dako, M0744) and the number of BrdU+ cells per intestinal crypt were counted from all well-oriented crypts present in 4 or 5 pictures. For cell migration, the distance between the start of the villus and the foremost BrdU+ enterocyte was measured. For mucus layer visualization, intestines were fixed in Carnoy’s fixative for 7 d. Sections were stained using Alcian blue/periodic acid–Schiff at pH 1.5. 16S FISH was performed using a mix of Cy-3 labeled probes targeting eubacteria (Supplementary Table S2).^40^ Pictures were made using a Confocal LSM 980 Airyscan 2 (Zeiss) with 20x magnification.

### Crypt isolation and MSI analysis

Small intestinal crypts were isolated and cultured in 10% R-Spondin conditioned medium and 10% Noggin conditioned medium supplemented with 250 nM 6-TG to select for MSH2-deficient crypts.^41,42^ Individual organoids were transferred into DirectPCR Lysis Reagent (Viagen) containing 0.5 mg/ml Proteinase K (Sigma). MSI analysis was conducted at mBat26 and mBat37 loci.^43^ Samples were run on a 3500xL Genetic Analyzer (Applied Biosystems). WT allele size was determined using tail DNA. Both markers were scored for the presence (1) or absence (0) of slippage, resulting in a cumulative MSI score ranging from 0 to 2 per individual organoid.^44^

### Statistical analysis

Data analyses were performed using GraphPad Prism (version 8). Data show means ± SD unless stated otherwise. The statistical tests used are described in figure legends. For comparison of two groups of continuous, normally distributed data, Student’s t-test was used. For comparison of a single variable between multiple groups of normally distributed continuous data, we used one-way ANOVA, followed by Tukey’s post-hoc analyses. For comparison of ≥2 variables between multiple groups, two-way ANOVA was used, with Sidak’s post-hoc analysis. Fisher’s exact test (two groups) or Chi-square test (>2 groups) was used to assess significant differences between categorical variables; for post-hoc analyses of Chi-square test, separate groups were analyzed by Fisher’s exact test and Bonferroni correction was applied. All tests were performed two-tailed. P-values <0.05 were considered statistically significant. Sample sizes for mouse intervention experiments were predetermined using G*Power software (version 3.1). * P < .05, ** P < .01, *** P < .001, **** P < .0001.

### Standard methods

Standard flow cytometry, genomic and transcriptomic analyses are provided as supplementary material.

## Supplementary Material

Supplemental Material

## Figures and Tables

**Table:** 

APC	Adenomatous polyposis coli
BrdU	5-bromo-2’-deoxyuridine
BSA	Bovine serum albumin
DTT	1,4-Dithiothreitol
EGFR	epidermal growth factor receptor
FBS	Fetal bovine serum
FFPE	formalin-fixed paraffin embedded
FISH	fluorescent in situ hybridization
FMT	Fecal microbiota transplantation
GSEA	Gene set enrichment analysis
IPA	Ingenuity pathway analysis
LPL	Lamina propria lymphocyte
LS	Lynch syndrome
MLH1	MutL homologue 1
MMR	DNA mismatch repair
MSH2	MutS homologue 2
MSH6	MutS homologue 6
PCoA	Principal component analysis
PMS2	Postmeiotic segregation increased 2
SPF	Specific-pathogen-free
TMZ	Temozolomide

## Data Availability

**Transcript profiling**: RNA sequencing data have been deposited at the National Center for Biotechnology Information, under the accession number GSE171996. **Microbiome analyses**: 16S rRNA microbiome sequencing data is available at https://www.ebi.ac.uk/ena/browser/view/PRJEB45308
