# Supplementary material for: Pro-mutagenic effects of the gut microbiota in a Lynch syndrome mouse model
Source: Gut Microbes. 2022 Feb 21;14(1):2035660. doi: 10.1080/19490976.2022.2035660 (PMC8865281; doi:10.1080/19490976.2022.2035660)
Supplement: Supplemental Material [file KGMI_A_2035660_SM5342.zip › supplementary/Supplementary Data.docx]

**
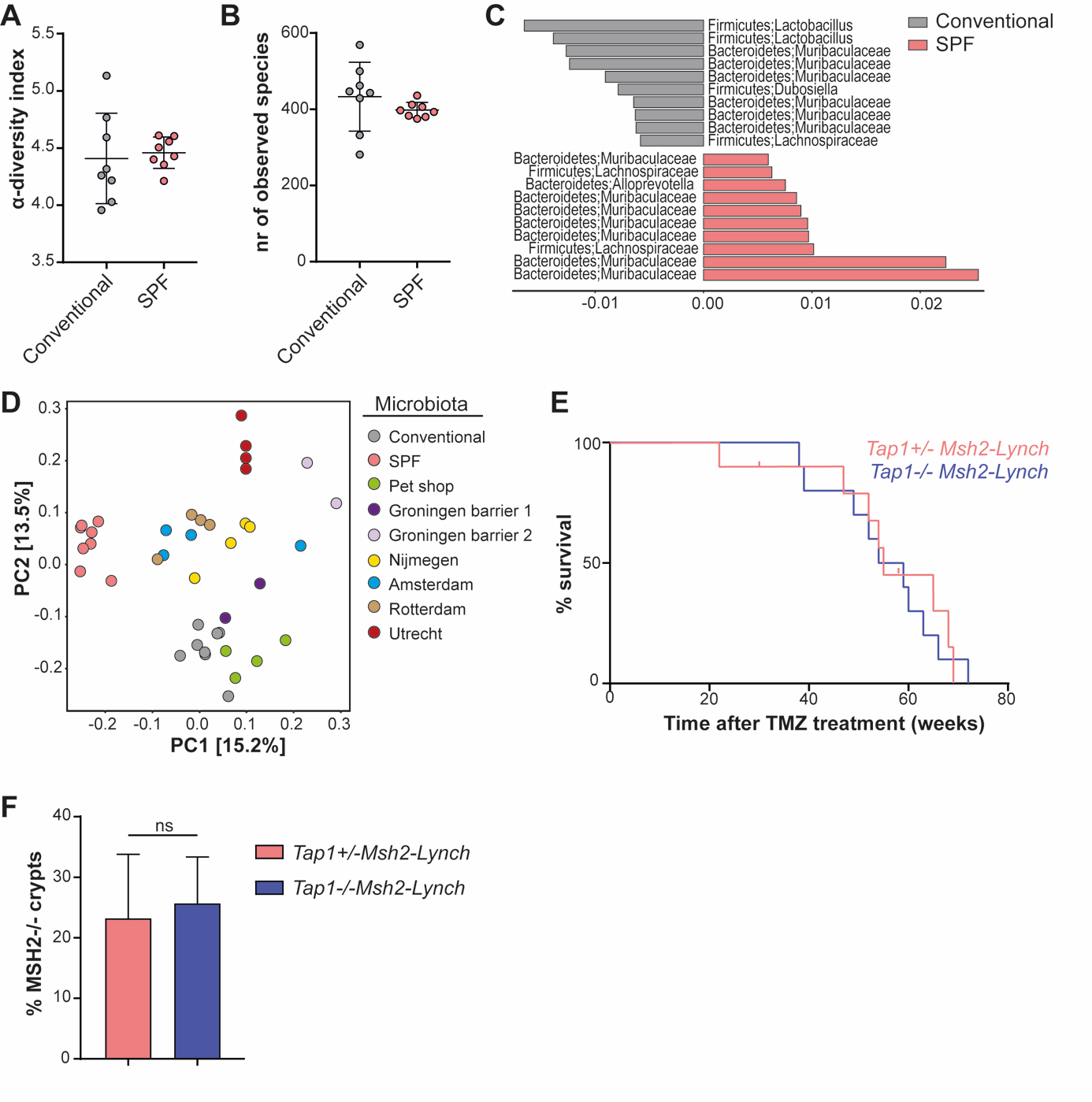
SUPPLEMENTARY FIGURE S1**

**Supplementary Figure S1. A,** Shannon index for α-diversity. **B,** Number of observed bacterial species. **C,** Top 10 bacterial taxa that showed significantly altered relative abundances between conventional and SPF mice. Significance was determined using Permutational multivariate analysis of variance (PERMANOVA). **D,** Unweighted UniFrac PCoA comparing the fecal microbiota composition of SPF and conventional *Msh2-Lynch* mice to microbiotas derived from conventional mice housed in other Dutch animal facilities and from a pet shop, as determined by 16S rRNA gene profiling. **E,** Survival of SPF *Tap1*^-/-^ *Msh2-Lynch* and *Tap1^+/-^ Msh2-Lynch* mice after 5d TMZ treatment. **F,** Quantification of the percentage of MSH2-deficient crypts in the intestinal epithelium of *Tap1*^-/-^ *Msh2-Lynch* (n=10) and *Tap1^+/-^ Msh2-Lynch* mice (n=8) after TMZ treatment, as measured from two fields of view. Plotted are mean and SD. P values were calculated using the Student’s t test. ns indicates a significance level of P>0.05.

**SUPPLEMENTARY FIGURE S2**

**
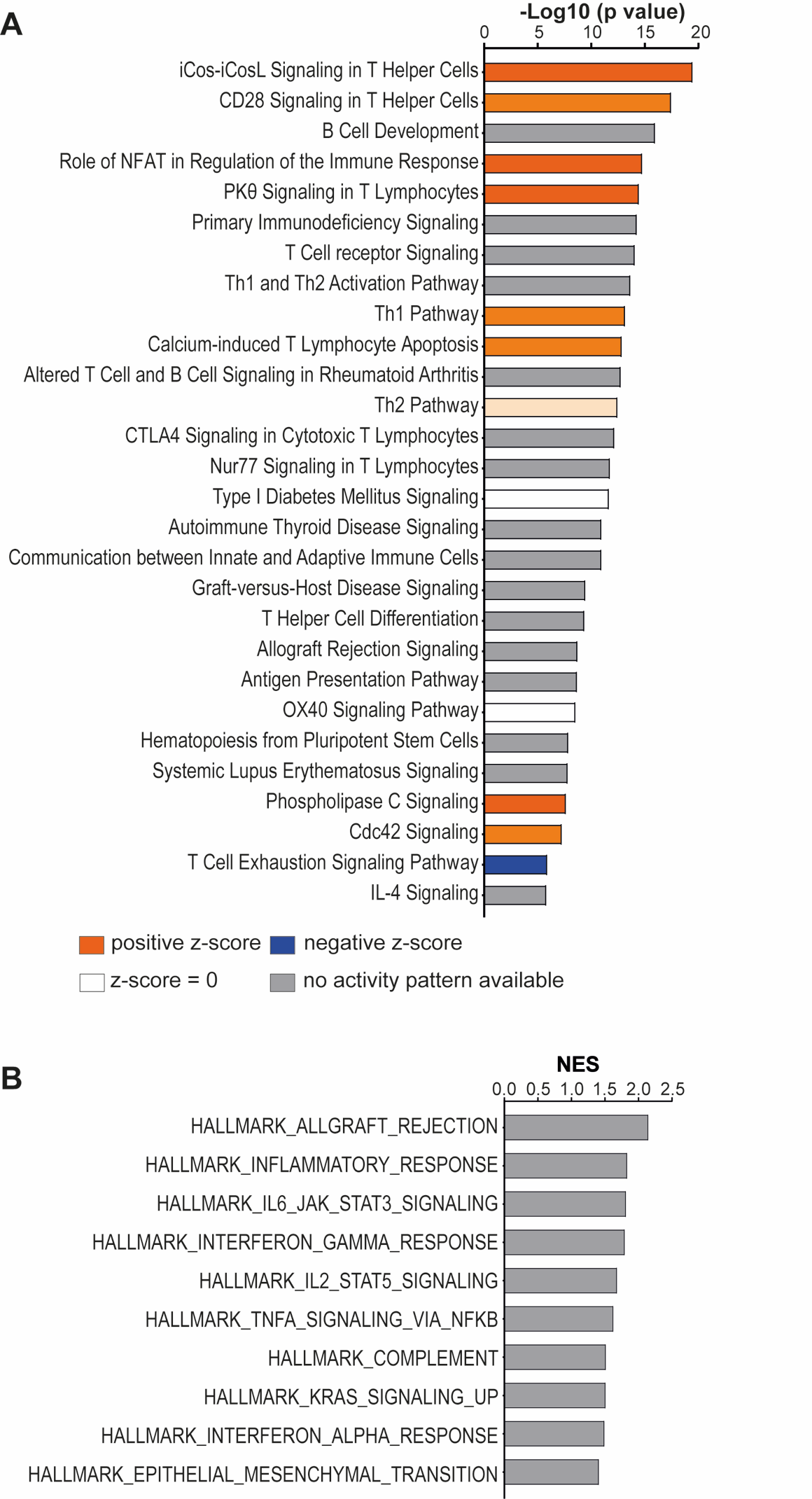
**

**Supplementary Figure S2. A,** IPA on differentially expressed genes between SPF and conventional mice. Top 28 of differentially activated pathways are shown. **B,** GSEA comparing transcriptional profiles of conventional mice and SPF mice using MSigDB Hallmark gene sets. Normalized enrichment score (NES) and false discovery rate (FDR) are indicated. Plotted are all Hallmark gene sets that showed enrichment in conventional mice with FDR q-value <0.05.

**SUPPLEMENTARY FIGURE S3**

**
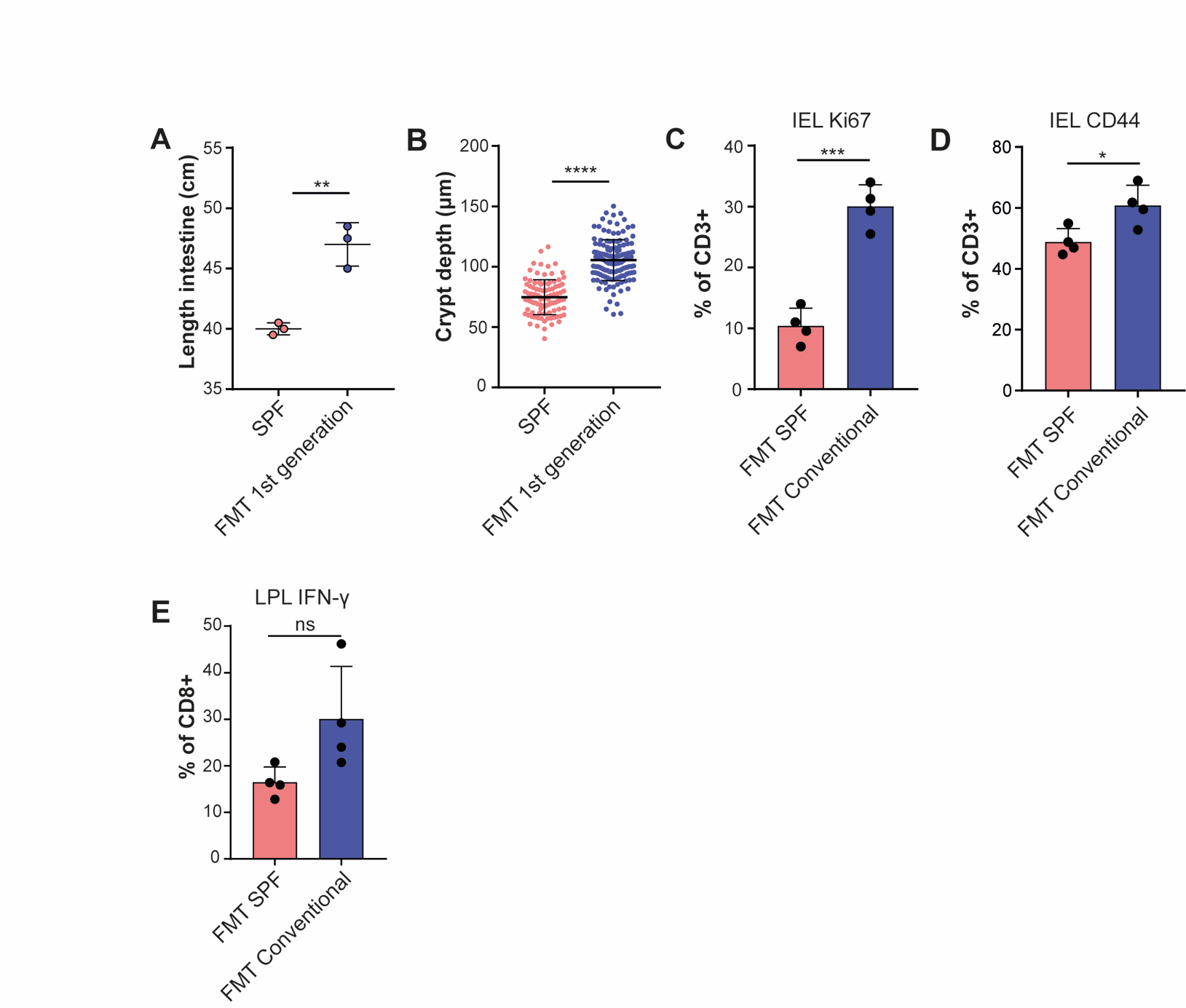
**

**Supplementary Figure S3. A,** Quantification of small intestinal length of mice born in SPF conditions or as the first generation after FMT (n=3). **B,** Quantification of small intestinal crypt depth as a measure for intestinal proliferation in mice born in SPF conditions or as the first generation after FMT (n=3). **C,** Frequency of Ki67+ T cells in IEL upon SPF or conventional FMT, gated on live, CD45+, CD3+ T cells. **D,** Frequency of CD44+ T cells in IEL upon SPF or conventional FMT, gated on live, CD45+, CD3+ T cells. **E,** Frequency of IFN-γ+ T cells in IEL upon SPF or conventional FMT, gated on live, CD45+, CD3+ T cells.

**SUPPLEMENTARY FIGURE S4**

**
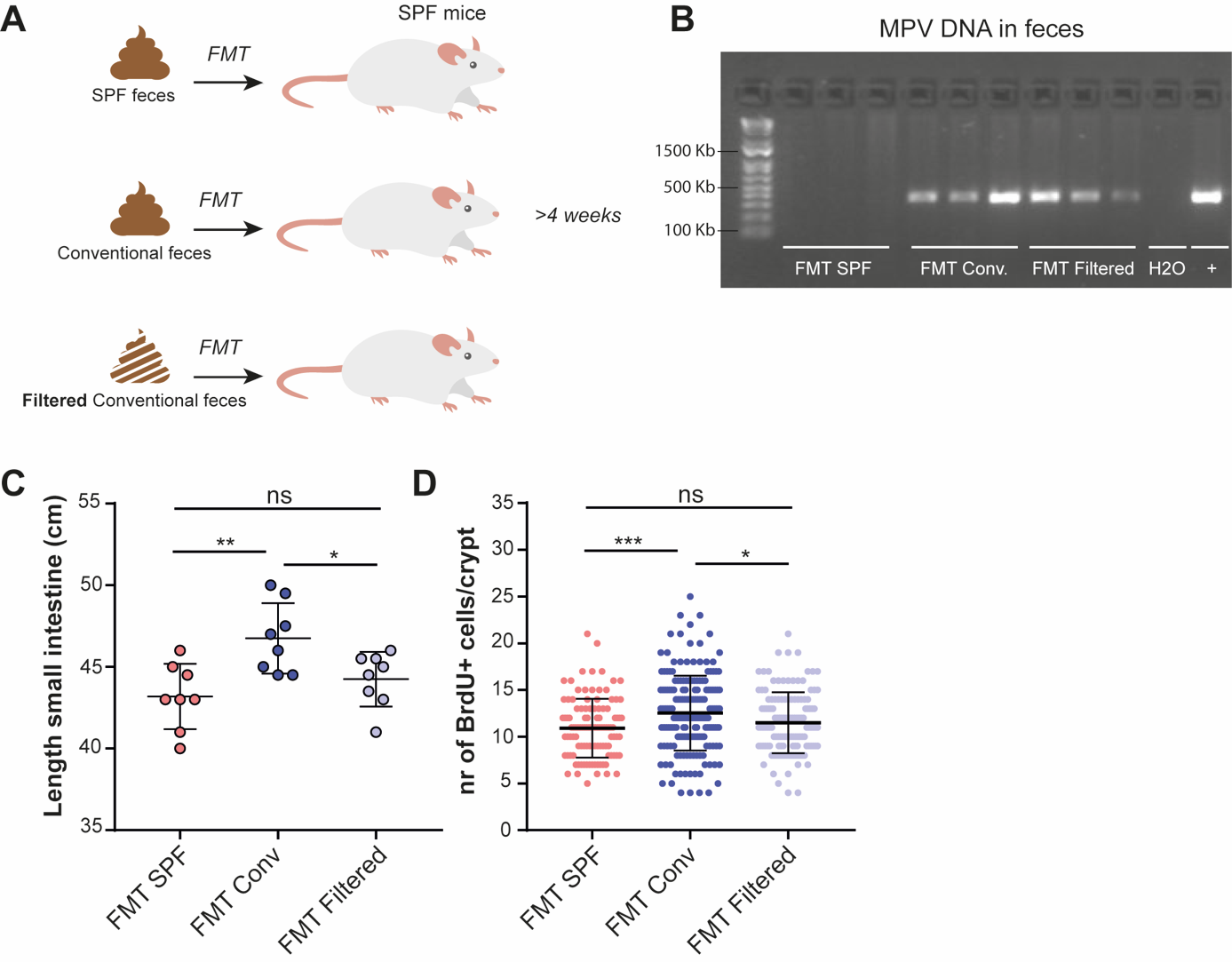
**

**Supplementary Figure S4. A,** Experimental setup. SPF mice received FMT with suspension of -80°C preserved SPF, conventional or 0.22 µm filter sterilized conventional feces. **B,** Picture of 2% agarose gel showing MPV-specific PCR products. DNA extracted from a lymph node of an MPV infected mouse was used as a positive control **C,** Quantification of small intestinal length after FMT with SPF, conventional or filtered conventional fecal suspension (n=8). **D,** Quantification of BrdU-positive cells per crypt in jejunal tissue sections from mice that received FMT with SPF (n=7), conventional (n=8) or filtered conventional (n=8) fecal suspension. Plotted are mean and SD. P values were calculated using a One-way ANOVA, using Tuckey’s test for multiple comparisons. Asterisks indicate a significance level of P<0.05, P<0.01, P<0.001 and P<0.0001, respectively.

**SUPPLEMENTARY FIGURE S5**

**
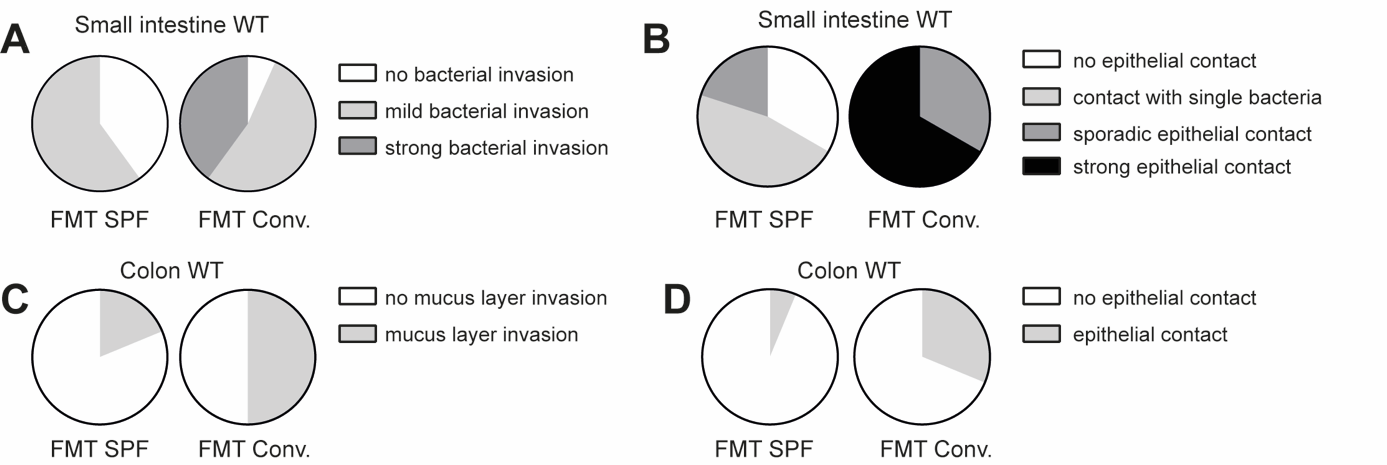
**

**Supplementary Figure S5**. **A,** Scoring of 16S rRNA FISH images for bacterial presence between intestinal villi in WT mice at 4 weeks after SPF or conventional FMT. **B,** Scoring of bacterial contact with the small intestinal epithelium. **C,** Scoring of 16S rRNA FISH images for bacterial invasion into the colonic mucus layer in WT mice. **D,** Scoring of bacterial contact with the colonic epithelium.

**SUPPLEMENTARY FIGURE S6**

**
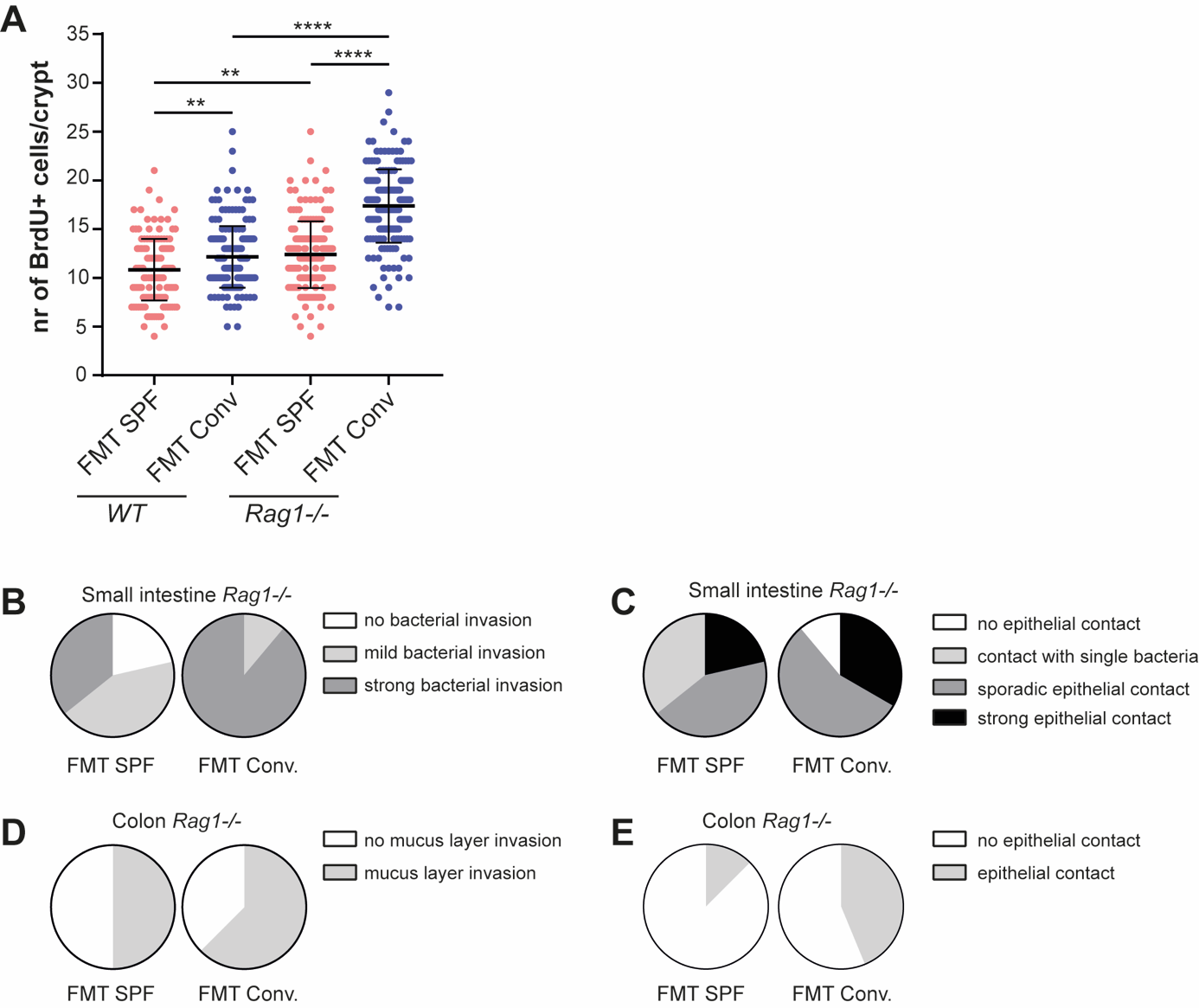
**

**Supplementary Figure S6**. **A,** Quantification of BrdU-positive cells per crypt in jejunal tissue sections from *WT* and *Rag1^-/-^* mice (n=4) that received FMT with SPF or conventional FMT. Plotted are mean and SD. P values were calculated using a One-way ANOVA, using Tuckey’s test for multiple comparisons. Asterisks indicate a significance level of P<0.05, P<0.01, and P<0.001, respectively. **B,** Scoring of 16S rRNA FISH images for bacterial presence between intestinal villi in *Rag1^-/-^* mice at 4 weeks after SPF or conventional FMT. **C,** Scoring of bacterial contact with the small intestinal epithelium. **D,** Scoring of 16S rRNA FISH images for bacterial invasion into the colonic mucus layer in *Rag1^-/-^* mice. **E,** Scoring of bacterial contact with the colonic epithelium.

**SUPPLEMENTARY TABLE S1.** **Overview of excluded pathogens in SPF mice.**

| **Agent** | **Material** |
| --- | --- |
| **PCR** |  |
| Minute virus of mice (MVM) | Feces |
| Mouse Hepatitis virus (MHV) | Feces |
| Mouse parvovirus (MPV) | Feces |
| Epizootic diarrhea of infant mice virus (EDIM) | Feces |
| Mouse norovirus (MNV) | Feces |
| Theiler's encephalomyelitis virus (TMEV) | Feces |
| Mouse adenovirus 1 (MAD1) | Feces |
| Mouse adenovirus 2 (MAD2) | Feces |
| *M. pulmonis* | Dry oral swab |
| *C. piliforme* | Feces |
| *Helicobacter* | Feces |
| *P. pneumotropica* | Dry oral swab |
| *S. penumoniae* | Dry oral swab |
| *Salmonella* spp. | Feces |
| *S . moniliformis* | Dry oral swab |
| *Staphylococcus aureus* | Dry oral swab |
| *C. rodentium* | Feces |
| *C. kutscheri* | Dry oral swab |
| Pinworms (*S. obvelata, S. muris, A . tetraptera*) | Feces |
| Fur mites (*Myocoptes, Myobia, Radforia*) | Fur Swab |
| Streptococci b-haemolytic (Group A) | Dry oral swab |
| Streptococci b-haemolytic (Group B) | Dry oral swab |
| Streptococci b-haemolytic (Group C) | Dry oral swab |
| Streptococci b-haemolytic (Group G) | Dry oral swab |
| *Corynebacterium bovis* | Fur swab/Feces |
| Mouse kidney parvovirus (MKPV) | Feces |
| Dermatophytes | Fur/Skin scrape |
| **Serology** |  |
| Lymphocytic choriomeningitis virus (LCMV) | Opti-Spot |
| Ectromelia virus (ECTV) | Opti-Spot |
| Pneumonia virus of mice (PVM) | Opti-Spot |
| Reovirus type 3 (Re03) | Opti-Spot |
| Sendai virus (SeV) | Opti-Spot |
| MHV | Opti-Spot |
| MNV | Opti-Spot |
| MPV | Opti-Spot |
| MVM | Opti-Spot |
| EDIM | Opti-Spot |

| Reagents |  |
| --- | --- |
| **Antibodies for flow cytometry** |  |
| Armenian Hamster anti-CD3e, FITC (clone 145-2C11) | Biolegend |
| Armenian Hamster anti-CD4, BUV805 (clone GK 1.5) | BD Biosciences |
| Rat anti-CD8, AF700 (clone 56-6.7) | Biolegend |
| Rat anti-CD44, BV605 (clone IM7) | Biolegend |
| Rat anti-CD45, BUV395 (clone 30F11) | BD Biosciences |
| Rat anti-Ki67, PerCP-eF710 (clone SolA15)) | eBioscience |
| Armenian Hamster anti-TCRγδ, (clone GL-3) | Biolegend |
| Rat anti-IFNγ-, PE-Cy7 (clone XMG1.2) | Biolegend |
| Rat anti-IL17a, PE (clone eBio17B7) | eBioscience |
| Fixable Viability Dye eFluor 780 | eBioscience |
| **Primers 5'-3'** |  |
| MPV FW | ACTGCTTCAATGATGGTTGCTC |
| MPV RV | CTGACCCAGCTGCCTGTTTG |
| **qPCR primers 5'-3'** |  |
| *Areg* FW | GGTCTTAGGCTCAGGCCATTA |
| *Areg* RV | CGCTTATGGTGGAAACCTCTC |
| *Ctnnb1* FW | ATGGAGCCGGACAGAAAAGC |
| *Ctnnb1* RV | CTTGCCACTCAGGGAAGGA |
| *Gapdh* FW | AGGTCGGTGTGAACGGATTTG |
| *Gapdh* RV | TGTAGACCATGTAGTTGAGGTCA |
| *Lgr5* FW | CCTACTCGAAGACTTACCCAGT |
| *Lgr5* RV | GCATTGGGGTGAATGATAGCA |
| *Muc2* FW | ACCTGGGGTGACTTCCACT |
| *Muc2* RV | ACCTGGGGTGACTTCCACT |
| *Muc3* FW | GCCGTGAATTGTATGAACGGA |
| *Muc3* RV | CGCAGTTGACCACGTTGACTA |
| *Nod2* FW | CACACATGGCCTTTGGTTTCCAGT |
| *Nod2* RV | AAAGAGCTGCAGTTGAGGGAGGA |
| *Pla2g5* FW | GGCCTAGTCATCTGCGAACAC |
| *Pla2g5* RV | TGGGGTAATACTGGTAAAGAGGG |
| *Reg3a* FW | TCACCTGGTCCTCAACAGTATT |
| *Reg3a* RV | GGAGCGATAAGCCTTGTAACC |
| *Reg3b* FW | ATGCTGCTCTCCTGCCTGATG |
| *Reg3b* RV | CTAATGCGTGCGGAGGGTATATTC |
| *Reg3γ* FW | TTCCTGTCCTCCATGATCAAAA |
| *Reg3γ* RW | CATCCACCTCTGTTGGGTTCA |
| *Tlr2* FW | CGCCCTTTAAGCTGTGTCTC |
| *Tlr2* RV | CGATGGAATCGATGATGTTG |
| *Wnt3* FW | CTCGCTGGCTACCCAATTTG |
| *Wnt3* RV | CTTCACACCTTCTGCTACGCT |
| **16S FISH probes 5'-3'** |  |
| EUB338-I | Cy3-GCTGCCTCCCGTAGGAGT |
| EUB338-II | Cy3-GCAGCCACCCGTAGGTGT |
| EUB338-III | Cy3-GCTGCCACCCGTAGGTGT |
| Nonsense | Cy3-CGACGGAGGGCATCCTCA |

**SUPPLEMENTARY TABLE S2.** **Overview of reagents used in this study.**

**SUPPLEMENTARY TABLE S3.** **Pathogens identified in conventional, SPF and Conventional FMT mice by commercial pathogen screening.**

|  | Conventional | SPF | FMT Conventional |
| --- | --- | --- | --- |
| **Viruses** |  |  |  |
| Mouse hepatitis virus (MHV) | + | - | - |
| Mouse norovirus (MNV) | + | - | + |
| Mouse parvovirus (MPV) | + | - | + |
| **Bacteria** |  |  |  |
| *Actinobacillus muris* Like | + | - | - |
| *Corynebacterium* bovis | + | - | - |
| *Helicobacter* Spp. | + | - | + |
| *Helicobacter hepaticus* | + | - | + |
| *Pasteurella pneumotropica-Heyl* | + | - | - |
| *Pasteurella pneumotropica-Jawetz* | + | - | - |
| *Streptococcus* Sp. | + | - | - |
| **Parasites** |  |  |  |
| *Aspicularis tetraptera* | + | - | - |
| *Chilomastix* spp. | + | - | + |
| *Entamoeba* muris | + | - | + |
| *Giardia* | + | - | - |
| *Spironucleus* spp. | + | - | - |
| *Syphacia obvelata* | + | - | - |
| *Tritrichomonas muris* | + | - | + |

**SUPPLEMENTARY TABLE S4.** **Survival (weeks) and tumor phenotype in *Msh2-Lynch* mice.**

| Treatment | Case number | Survival after TMZ | Extraintestinal tumor | Intestinal pathology |
| --- | --- | --- | --- | --- |
| FMT SPF | none | 9 | lymphoid |  |
| FMT SPF | 20HTR210 | 10 | lymphoid |  |
| FMT SPF | 21HTR27 | 10 | lymphoid |  |
| FMT SPF | 21HTR35 | 12 |  |  |
| FMT SPF | 21HTR11 | 12 | lymphoid |  |
| FMT SPF | 21HTR37 | 13 | lymphoid |  |
| FMT SPF | 20HTR242 | 13 | lymphoid |  |
| FMT SPF | 21HTR52 | 23 | lymphoid |  |
| FMT SPF | * | 23 |  |  |
| FMT SPF | 21HTR72 | 26 | lymphoid |  |
| FMT SPF | * | 30 |  |  |
| FMT SPF | 21HTR69 | 32 | lymphoid |  |
| FMT conv | 19HTR158 | 7 | lymphoid |  |
| FMT conv | 19HTR162 | 8 | lymphoid |  |
| FMT conv | 19HTR165 | 9 | lymphoid |  |
| FMT conv | 19HTR187 | 11 | lymphoid |  |
| FMT conv | 19HTR205 | 16 | lymphoid |  |
| FMT conv | 19HTR207 | 17 | lymphoid |  |
| FMT conv | 20HTR24 | 27 | lymphoid |  |
| FMT conv | 20HTR97 | 39 | lung |  |
| FMT conv | 20HTR108 | 41 | lung |  |
| FMT conv | 20HTR110 | 42 | lung |  |
| conv 1st gen | 20HTR20 | 7 |  |  |
| conv 1st gen | 25HTR25 | 9 | lymphoid |  |
| conv 1st gen | 20HTR36 | 11 | lymphoid |  |
| conv 1st gen | 20HTR54 | 15 | lymphoid |  |
| conv 1st gen | 20HTR55 | 16 | lymphoid |  |
| conv 1st gen | 20HTR116 | 29 | lung |  |
| conv 1st gen | 20HTR157 | 41 | lung | 4 |
| conv 1st gen | 20HTR228 | 51 | lung |  |

Survival (weeks) and histopathological analysis of *Msh2-Lynch* mice that received SPF or conventional FMT, or were born as a first generation after conventional FMT (FMT 1^st^ gen), after treatment with 10d TMZ. SPF: specific pathogen free; 1: focal hyperplasia; 2: gastrointestinal neoplasia (GIN), 3: adenoma; 4: adenocarcinoma.
