# Supplementary material for: Pro-mutagenic effects of the gut microbiota in a Lynch syndrome mouse model
Source: Gut Microbes. 2022 Feb 21;14(1):2035660. doi: 10.1080/19490976.2022.2035660 (PMC8865281; doi:10.1080/19490976.2022.2035660)
Supplement: Supplemental Material [file KGMI_A_2035660_SM5342.zip › supplementary/Supplementary Materials and Methods.docx]

**SUPPLEMENTARY MATERIALS AND METHODS**

**Flow cytometry**

Small intestines were flushed, defatted and Peyers’ patches were removed. Intestines were incubated in extraction medium containing 30 ml RPMI, 93 µl DTT (5%w/v), 60 µl 0.5M EDTA and 500 µl FBS for 15 min at 37 °C while shaking. The IEL cell fraction was filtered using 100 µm and 70 µm strainers, centrifuged (5 min, 1200 rpm) and resuspended in FACS buffer (PBS, 0.1% BSA, 2 mM EDTA, 10 mM HEPES). The remaining tissue was washed in cold RPMI, fragmented, and incubated for 30min in digestion medium containing 25 ml RPMI, 0.2U/ml Liberase™ TM (Roche) and 300 µl FBS at 37 °C while shaking. The LPL suspension was filtered using 100 µm and 70 µm strainers, centrifuged and resuspended in FACS buffer. Stimulation and staining occurred as described previously^1^. Antibodies are listed in Supplementary Table 2. Experiments were performed on a LSR-II flow cytometer (BD Biosciences) using FACS-Diva software. Data was analyzed with FlowJo™ V10 software.

**qPCR and RNA sequencing**

Small intestines were retrieved from -80°C storage, crushed under cryogenic conditions and lysed in RLT buffer (Qiagen) supplemented with β-mercaptoethanol (Sigma). RNA was isolated using the RNeasy Mini Kit (Qiagen). cDNA generation and qPCR was performed as described^2^. Data were normalized against GAPDH expression. Primer sequences are listed in Supplementary Table 2. For RNA sequencing, strand-specific cDNA libraries were generated using the TruSeq Stranded mRNA sample preparation kit (Illumina). Libraries were sequenced as 65bp single reads on a HiSeq2500 (Illumina). Data was analysed using Qlucore Omics explorer 3.6. Gene set enrichment analysis was conducted as described^3^. Pathway enrichment analysis was performed using Ingenuity Pathway Analysis (IPA) software (Qiagen).

**Whole exome sequencing**

Tumor DNA was isolated from formalin fixed paraffin embedded (FFPE) tissues using the QIAamp DNA FFPE Tissue Kit (Qiagen). Tail or ear DNA was used as a normal control. Libraries were prepared using the KAPA HTP Prep Kit (KAPA Biosystems). Sureselect exome target enriched samples were sequenced paired-end on the Hiseq 2500 (Illumina) high-out modus. Raw reads were adapterclipped using cutadapt, and aligned to the mouse genome (GRCm38). Somatic variants were called with mutect2 and annotated using SNPeff, and SNPsift^4,5^.

1. Wellenstein MD, Coffelt SB, Duits DEM, et al. Loss of p53 triggers WNT-dependent systemic inflammation to drive breast cancer metastasis. Nature 2019;572:538–542.

2. Benedict B, Schie JJM van, Oostra AB, et al. WAPL-Dependent Repair of Damaged DNA Replication Forks Underlies Oncogene-Induced Loss of Sister Chromatid Cohesion. Dev Cell 2020;52:683–698.e7.

3. Subramanian A, Tamayo P, Mootha VK, et al. Gene set enrichment analysis: A knowledge-based approach for interpreting genome-wide expression profiles. Proc Natl Acad Sci U S A 2005;102:15545–15550.

4. Cingolani P, Patel VM, Coon M, et al. Using Drosophila melanogaster as a model for genotoxic chemical mutational studies with a new program, SnpSift. Front Genet 2012;3:1–9.

5. Cingolani P, Platts A, Wang LL, et al. A program for annotating and predicting the effects of single nucleotide polymorphisms, SnpEff. Fly (Austin) 2012;6:80–92.
